# Ca2+ imaging of self and other in medial prefrontal cortex during social dominance interactions in a tube test

**DOI:** 10.1073/pnas.2107942119

**Published:** 2022-07-26

**Authors:** Nuria Garcia-Font, Rufus Mitchell-Heggs, Kapil Saxena, Carolin Gabbert, Georgina Taylor, Giulia Mastroberardino, Patrick A. Spooner, Francesco Gobbo, Julia K. Dabrowska, Sumantra Chattarji, Peter C. Kind, Simon R. Schultz, Richard G. M. Morris

**Affiliations:** ^a^Laboratory for Cognitive Neuroscience, Edinburgh Neuroscience, The University of Edinburgh, Edinburgh EH8 9JZ, United Kingdom;; ^b^Simons Initiative for the Developing Brain and Patrick Wild Centre, The University of Edinburgh, Edinburgh EH8 9JZ, United Kingdom;; ^c^Institute of Neurogenetics, University of Lübeck, 23562 Lübeck, Germany;; ^d^Department of Bioengineering and Centre for Neurotechnology, Imperial College London, London SW7 2AZ, United Kingdom;; ^e^National Centre for Biological Sciences, Tata Institute of Fundamental Research, 400005 Bangalore, India

**Keywords:** social dominance, tube test, endoscopic imaging, prefrontal cortex, rodents

## Abstract

The study of social dominance interactions between animals offers a window onto the decision-making involved in establishing dominance hierarchies and an opportunity to examine changes in social behavior observed in certain neurogenetic disorders. Competitive social interactions, such as in the widely used tube test, reflect this decision-making. Previous studies have focused on the different patterns of behavior seen in the dominant and submissive animal, neural correlates of effortful behavior believed to mediate the outcome of such encounters, and interbrain correlations of neural activity. Using a rigorous mutual information criterion, we now report that neural responses recorded with endoscopic calcium imaging in the prelimbic zone of the medial prefrontal cortex show unique correlations to specific dominance-related behaviors. Interanimal analyses revealed cell/behavior correlations that are primarily with an animal’s own behavior or with the other animal’s behavior, or the coincident behavior of both animals (such as pushing by one and resisting by the other). The comparison of unique and coincident cells helps to disentangle cell firing that reflects an animal’s own or the other’s specific behavior from situations reflecting conjoint action. These correlates point to a more cognitive rather than a solely behavioral dimension of social interactions that needs to be considered in the design of neurobiological studies of social behavior. These could prove useful in studies of disorders affecting social recognition and social engagement, and the treatment of disorders of social interaction.

## Significance

Growing interest in social neuroscience requires the development and refinement of social behavior paradigms and novel analyses of the manner in which the neural activity in one brain may reflect the behavioral activity of self or of other, or the activity of each of two interacting individuals. This study documents an approach to analyze such interactions in a social dominance encounter between rodents.

The overarching aim of this work is to develop a better understanding of the neural activity associated with social dominance interactions, including those that may be altered in autism spectrum disorders (ASDs). Social interactions occur between pairs of rodents taking part in a variety of social tests used in laboratory settings (1, 2), including tests of relative access to food, resident–intruder tests, and the widely used “tube test” of social dominance (3, 4). The latter takes the form of two animals being placed into a Plexiglas tube, confronting each other during an interanimal encounter that is resolved by one animal becoming the “winner” and the other animal the “loser.” Sometimes, this involves the behavioral conjunction of “pushing” by one and “resisting” by the other, but diverse other behaviors are observed that reflect a more cognitive element of decision-making, such as “withdrawal.” Extensive use of the tube test in group-living male mice has revealed it to be a stable and reliable indicator of social dominance, correlating well with other measures of dominance (2, 5). The present study is part of a larger project to use this and other tests of phenotype in animal models of ASDs because a prominent characteristic of this syndrome is social withdrawal. For example, Saxena et al. (6) showed, using wild-type (WT) and Fragile-X mutant (*Fmr1*^-/y^) rats, that repeated testing over several sessions revealed a relative but not absolute social dominance of WT animals over the mutants, with gene-related differences in intersession stability. We also witnessed the development of inflexible behavioral habits that enabled overcoming failures of social awareness in some encounters. These findings are consistent with the socially withdrawn or otherwise aberrant behavior seen in humans with ASD and in animal models of Fragile-X (7) and Rett syndrome (8, 9).

In this study, we examined neural correlates of social dominance interactions from a cognitive perspective. It was guided by anatomical and physiological data revealing that the frequency of excitatory postsynaptic currents, single-unit activity, or Ca^2+^ transients in the prefrontal cortex or anterior cingulate gyrus are likely very important mediators of dominance and known to be on the causal chain of brain network activity mediating dominance and submissiveness (10). Behavior by both animals, such as pushing or resisting, has been reported that is well correlated with changes in neural activity in the prelimbic zone of the medial prefrontal cortex (PrL, mPFC) of mice (5, 11). We have successfully identified behaviors such as moving forward, pushing, resisting, retreat, stillness, and withdrawal. Pushing and resisting may seem similar (and are sometimes conflated) but can be readily distinguished from the video record monitored on a frame-by-frame basis at 1/20 s by a skilled observer successfully identifying the initiating animal. In rats, the typical duration of tube test interactions varies from 5 s to 60 s. Encounters can be characterized as a cycle of specific acts, followed by perception of the immediate outcome of these actions by both animals. A dominant animal cannot just unilaterally “decide” to be dominant—it may need to push to find out how the other animal will react. Such interactions fuel the interbrain correlations between two animals outlined by ref. 12. Accordingly, while a focus on overt behaviors that can be scored from a video record is clearly objective, a strictly “behaviorist” approach risks failing to provide unambiguous insight into the underlying decision-making process(es) involved in ascertaining social dominance. Studies of sociability and social recognition memory also point to cognitive aspects of social interaction (13).

A separate issue is that the standard single-animal approach to examining the neural correlates of sensation, perception, and action is to look only at the animal from which physiological recordings are being taken (14–17). A social situation, however, opens up the intriguing possibility that the activity in the brain of one animal reflects a representation of the actions or intentions of the other animal. It rapidly became apparent to us that, while differences in single-cell or multiunit activity associated with such dominance or submissive behaviors were observed, as reported previously by Hu’s group (10), a neural correlate of pushing behavior by one animal could sometimes be described as the neural correlate of resistance by the other animal. Our study was conducted in the spirit of the interbrain approach, but our findings open up a separate logical issue from the concept of interbrain dynamics (12). Specifically, they include the possibility of identifying whether cell firing in animal A during encounters with animal B is specifically responsive to the actions of animal A, or of animal B, or of them both.

## Results

The data take the form of Ca^2+^ imaging data, behavioral data, and the analysis of mutual information between these. We report here data from three pairs of group-living WT rats, and three pairs of group-living FXS (Fmr1-/y) rats (*n* = 12). These are part of an ongoing study that will grow to much larger groups of animals over several years, sufficient to make detailed WT vs. FXS comparisons (not yet feasible). A total of 913 cells were identified from 11 of these animals, of which five pairs were analyzed in detail. We outline the protocol and histochemical analyses, then turn to quantification of the behavior, to the global ΔF/F measure, and, finally, using event processing software, to the patterns of events across multiple individual cells.

**Fig. 1.**
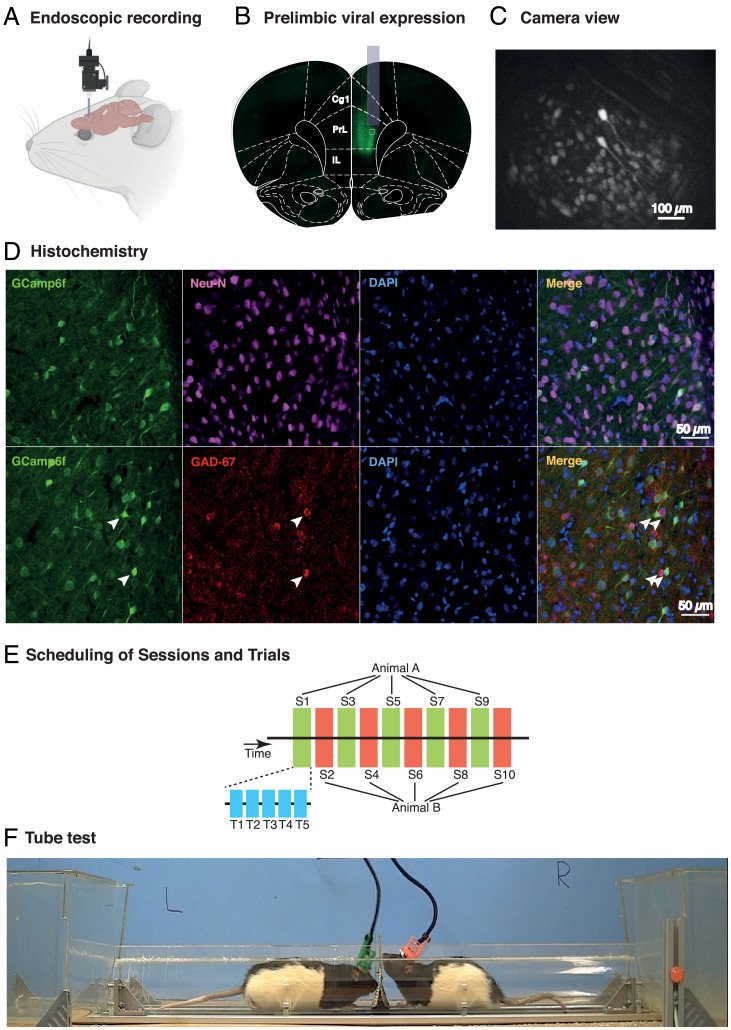
Calcium imaging in rats during social dominance interactions in the tube test. (A) Cartoon depicting detachable miniature endoscope (Inscopix) that is placed daily into a head-mounted baseplate cemented to the skull. (*B*) Photomicrograph of viral expression of GCamp6f colocalized on a coronal section from Paxinos and Watson brain atlas at the AP location of the prelimbic zone (PrL) of mPFC, together with superimposed image of typical location of a GRIN lens. (*C*) Representative camera view of PrL neurons during a behavioral session (Scale bar, 100 μm. based on the size of the sensor provided by Inscopix (1050x650μm equivalent to 1280x800pixels). (*D*) Higher-power images of GCamp6f expression in single cells (*Left*), Neu-N staining (*Left Middle Top*) and GAD-67 (*Left Middle Bottom*), DAPI (*Right Middle*), and exemplar superpositions (*Right*). (Scale bar, 50 μm.) (*E*) Recordings were taken from the two animals on alternate sessions in the tube test experiments consisting of five trials per session. With only one camera, recordings were taken from odd-numbered sessions for one animal and even-numbered sessions for the other. (*F*) Two Lister-hooded male rats in the tube test, each wearing the iHELMET that serves to protect the endoscopic from physical interaction with the other animal (18).

Fig. 1 *A–C* shows a cartoon of the placement of a microendoscope on the head of a hooded rat, representative viral expression of GCamp6f in the prelimbic area of the prefrontal cortex, and the approximate position of the gradient refractive-index (GRIN) lens in one of the rats, and a typical camera image during recording. Fig. 1*D* shows successful expression of GCamp6f in the PrL at cellular magnification (Fig. 1 *D*, *Left*; 20×). NeuN and GAD67 expression (Fig. 1 *D*, *Left Middle*), DAPI (Fig. 1 *D*, *Right Middle*), and exemplars of superposition (Fig. 1 *D*, *Right*) show that virus expression was in excitatory neurons (identified with NeuN and GAD67) without affecting nuclear integrity (observed with DAPI). Fig. 1*E* shows the protocols consisting of 50 encounters between the six pairs of animals over 10 d of tube tests, each having five trials per day (blue shading), of which alternate days (green, red) were subject to Ca^2+^ imaging between the animals of a pair. Fig. 1*F* shows two animals in the Plexiglas tube (1 m), one animal (green) having the real camera and the other (red) having a dummy camera, both protected within iHELMETs (18).

### Behavioral Findings.

**Fig. 2.**
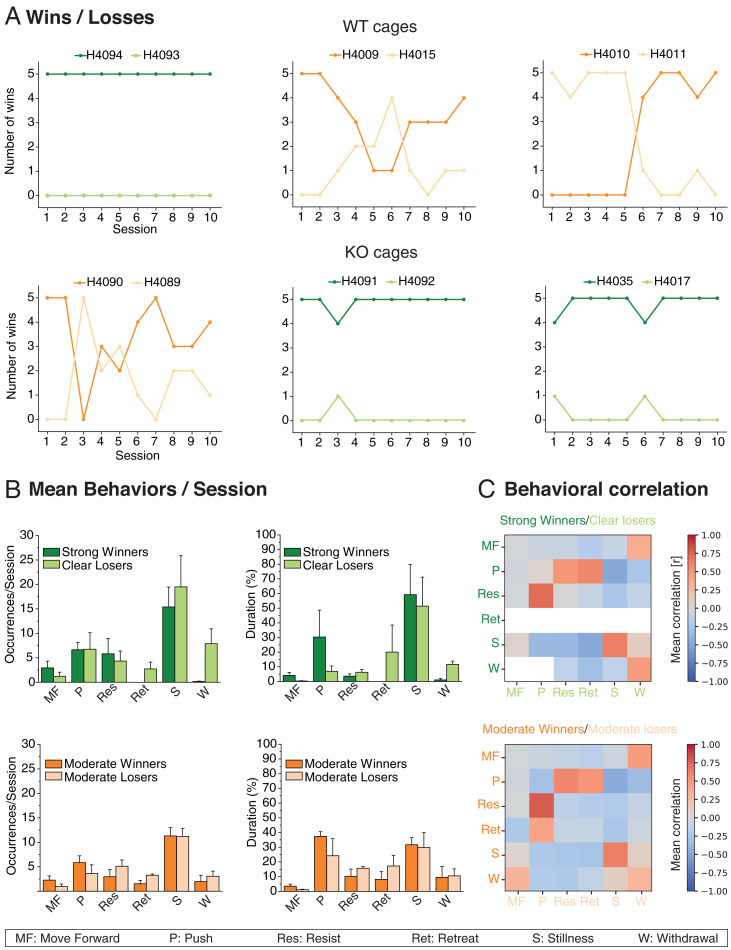
Behavioral analysis. (*A*) Timeline across 10 sessions of the six pairs of animals—three WT and three FXS. Note the strong dominance in three pairs of animals but a more varied and moderate pattern of dominance in the other three pairs. (*B*) Number of occurrences of behaviors per five trial sessions over 10 sessions averaged for the three strongly dominant cages (*Top*, green) and the three more moderate dominance cages (*Bottom*, orange). Note predominance of periods of STILLNESS, that RETREAT and WITHDRAWAL were not seen in the strongly dominant animals, and that there was a more uniform pattern across winners and losers in the case of moderate dominance. (*C*) Correlation matrix showing probability of behavior of animal A as a function of the cooccurrence of different behaviors in animal B (and vice versa). Note high PUSH/RESIST correlations, and absence of RETREAT and WITHDRAWAL in strongly dominant animals. Means ±1 SEM.

Fig. 2*A* shows the timelines of all pairs of animals in the tube test. In the cages of WT rats (Fig. 2 *A*, *Top*), one pair of animals displayed consistently strong dominance (H4093/H4094, dark and light green) while the other two pairs showed a more varied pattern of moderate dominance (dark and light orange); in the KO cages **(**Fig. 2 *A*, *Bottom*), two pairs of animals showed strong dominance, and only one pair showed a more moderate pattern (H4090/H4089). Different genotypes were hereafter pooled, but distinct dominance status retained.

Detailed frame-by-frame video analysis of the behaviors (Fig. 2*B*) revealed the behavioral profiles of the animals in each trial, plotted in terms of numbers of specific behaviors per session (averaged across 10 sessions), the relative duration of each type of behavior, and the interanimal behavior correlations (Fig. 2 *B* and *C*). The value of analysis at such fine temporal grain is that it is possible for a skilled observer to see clearly which of two animals initiates an encounter, that is, which animal first engages in PUSH behavior and which responds by RESIST or RETREAT behavior. Two of us (C.G. and G.M.) gained experience over 1,000 recorded trials of identifying these different behaviors. Fig. 2*B* considers pooled pairs of strong or moderate dominance pairs, with respect to both behavior frequency per session and normalized duration. We hypothesized that the “decision-making” in strong vs. moderate dominance pairs might be different. Excluding STILLNESS, an ANOVA of the frequency of occurrences in both strong and moderate pairs of animals showed a significant effect (Huyinh Feldt; F = 5.05, degree of freedom (df) 4/32, *P* = 0.003) but no significant difference between strong vs. moderate. For normalized duration, nonparametric Kruskal–Wallis tests comparing winners and losers showed that winners spent significantly longer time in MOVE FORWARD but shorter in RETREAT and WITHDRAWAL in the strongly dominant/submissive pairs (ps < 0.05, 0.037, and 0.05, respectively). In the more moderate dominance animals, only MOVE FORWARD showed a longer duration for the winners (*P* < 0.05).

In Fig. 2*C*, the correlation matrix shows the positive or negative correlations between specific behaviors of the winning animal in the series of paired encounters (rows) and those of the losing animal (columns). The color white implies that this combination of behaviors did not occur (generally, because one of the behaviors, such as RETREAT, did not occur in strong winners). RESIST behavior by a winning rat was strongly correlated with PUSH behavior by the losing animal, but, conversely, PUSH behavior by the winner correlated with both RESIST and RETREAT by the loser. This asymmetry reflects dominance. With respect to STILLNESS, there was a good but not unique correlation between the two animals. When one animal was STILL, it was likely that the other was also, but there were occasions when the winner was still and the losing animal showed WITHDRAWAL. WITHDRAWAL by the loser was also more strongly correlated with both MOVE FORWARD by the winner and, paradoxically, WITHDRAWAL by the winner. These behavioral correlations guided facets of the Ca^2+^ imaging analysis that follows.

### Mean Calcium Imaging Activity (ΔF/F) across All ROIs.

**Fig. 3.**
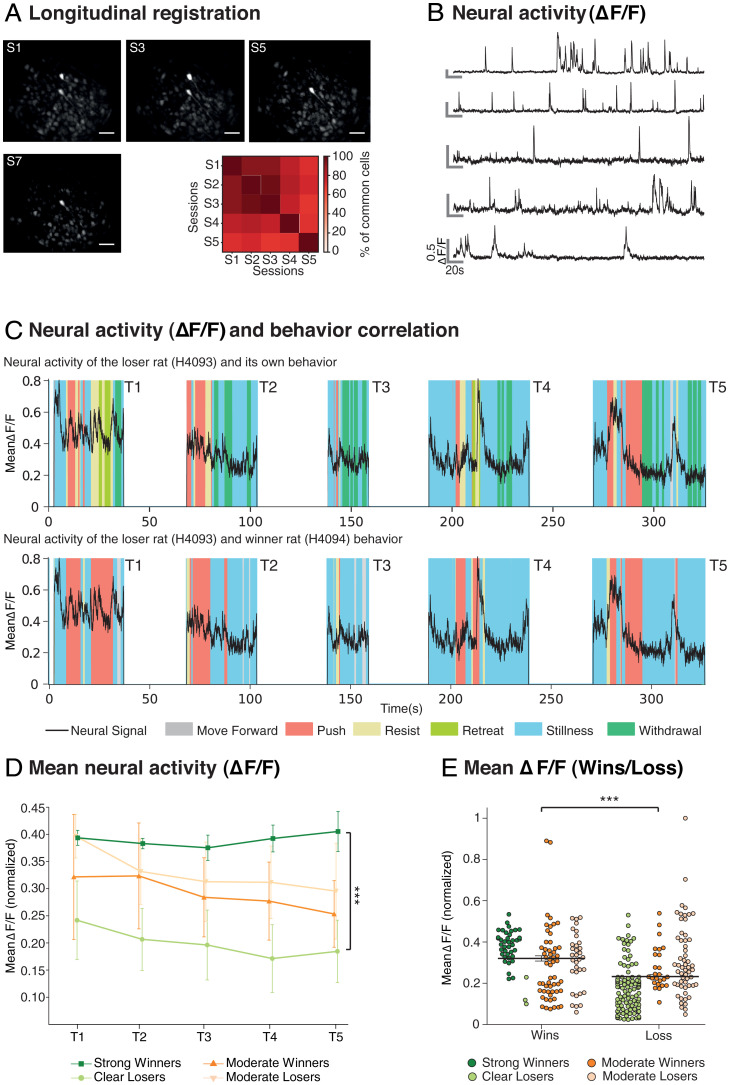
Neural activity in identified regions of interest. (*A*) Mean Longitudinal registration of the animals across five sessions (S1 to S5, *n* = 11) and exemplar of longitudinal registration of ROIs with respect to maximal fluorescence in individual animals within each session (Inscopix) across four sessions. ROIs are hereafter referred to as “cells.” Note the decline of likelihood of longitudinal registration across multiple sessions (Scale bar, 100 μm.). (*B*) Neural activity (ΔF/F) of an exemplar series of five individual cells showing the typical sharp rise time of luminance and slower decay time. These two parameters are conflated in any computation of mean neural activity. (*C*) Mean neural activity on each of five trials of a daily session aligned to an animal’s own behavior (H4093, *Top*) or to the observed behavior of the other animal (H4094, *Bottom*). Note the striking peak during PUSH and RESIST” (respectively), and sometimes during STILLNESS, as outlined in the text. Respective behaviors are color coded beneath the two images. (*D*) Normalized mean neural activity (ΔF/F) averaged across all cells and each of the five daily trials, categorized with respect to whether the animals were strongly dominant/clearly submissive or were displaying more moderate dominance (*n* = 11, ns = 2, 3, 3, and 3, respectively). A difference in mean neural activity is shown as a function of the strength of dominance. (*E*) Normalized mean neural activity (ΔF/F) averaged across all winning and losing trials. Note that it confirms that the mean ΔF/F is related to the strength of the dominance status (*n* = 11). (***P < 0.0001). Means ±1 SEM.

Successful stable recordings of Ca^2+^ transients from individual cells were secured (i.e., regions of interest [ROIs] showing multiple rapid increases in signal intensity of ΔF/F followed by slow decreases of fluorescence to baseline), with successful longitudinal registration gradually declining across successive sessions from >90% on successive session to >60% comparing session 1 (S1) and S5 (Fig. 3*A*). As we have only one endoscope in the laboratory, recordings were taken from one animal on even-numbered sessions but from the other on odd-numbered sessions. Using the CaImAn constrained nonnegative matrix factorization–extended (CNMF-e) (see *Materials and Methods* and Table 1 the total number of longitudinally registered cells identified from the 11 animals was 913 (WT = 375; KO = 538).

**Table 1.** Key resources

Reagent or Resource	Source	Identifier
Bacterial and virus strains		
AAV1.CamKII.GCaMP6f.WPRE.SV40	Addgene https://www.addgene.org/100834/#100834-AAV1	Cat#100834-AAV1
Deposited data		
Excel files of data	Laboratory	Attached
Experimental models: organisms/strains		
Rats (Male Long Evans-Fmr1^em1/PWC^) referred in text as Fmr1 KO	Breeders from SAGE Labs, now https://horizondiscovery.com/	In-house breading (The University of Edinburgh) Identifier: FMR1-HRB
Software and algorithms		
IDPS	https://www.inscopix.com/software-analyzis#software_idps	Version 1.5
CNMF-e	https://github.com/zhoupc/CNMF_E	Zhou et al. (28)
OASIS	https://github.com/j-friedrich/OASIS	Friedrich et al. (29)
MATLAB	https://www.mathworks.com/products/matlab.html	Version 2019b
SPSS	https://www.ibm.com/uk-en/products/spss-statistics	Version 25
ImageJ	NIH https://imagej.nih.gov/ij/index.html	Version 53
Other		
Inscopix Miniature Microscope system (nVista)	https://www.inscopix.com/nvista	nVista v3.0
Microinjection syringe pump	https://www.wpiinc.com/var-8091-microinjection-syringe-pump-with-smartouch-controller	Order code # UMP3T-1
GRIN lens, baseplate	https://www.inscopix.com/lenses-viruses	Lens (1.0 mm x 9.0 mm) Baseplate standard
Superbond (dental cement)	https://www.prestige-dental.co.uk/product/super-bond-universal-kit/	superbond-universal-kit
iHELMET (3D printed parts)	https://www.sciencedirect.com/science/article/pii/S0165027021000443	Saxena et al. (18). .STL files are in the supplementary for this
3D printer	https://www.makerbot.com/3d-printers/	PLA printer
Normal donkey serum	Sigma Aldrich	D9663
Triton X-100	Sigma Aldrich	T8787
Mouse Anti-GAD67	Sigma Aldrich	MAB5406 clone 1G10.2
Guinea pig Anti-NeuN	SYnaptic SYstems	266 004
Donkey anti-mouse Alexa 647	Thermo Fisher	A-31571
Goat Anti-Guinea pig IgG Cy3-conjugated	Abcam	ab102370
Fluoroshield with DAPI	Sigma Aldrich	F6057

The first step was to examine the neural activity of individual cells using the ΔF/F measure (Fig. 3*B*) and the summated mean across all cells in each animal, as in Kingsbury et al. (11), for each 1/20-s time period of the recorded tube test sessions (25 trials per rat; exemplar for one session of five trials in Fig. 3*C*). These sessions typically lasted 100 s to 350 s of which a large proportion was the intertrial interval when the animals were outside the Plexiglas tube (white; data from these periods was not analyzed). Time series plots of the within-tube normalized cell activity (black line, normalized scale on the *y* axis) reveal diverse patterns, including periods of quiescence, activity, and activity bursts (Fig. 3*C*). The different behaviors observed are color coded. The exemplar is a pair of rats (H4093 and H4094) in which the imaged animal was the loser of this series of contests. Trial 1 (T1) shows relative high activity and bouts of RESIST/PUSH/RESIST by the eventual loser (Fig. 3 *C*, *Top*), notably during two long PUSH periods by the winning competitor (Fig. 3 *C*, *Bottom*). Later in the same trial, PUSH by the winner is associated with RESIST/RETREAT and eventually WITHDRAWAL by the loser, with clearly detectable changes in the pattern of mean activity at any one time point. However, from T2 onward, it is quite striking how much time is spent by the winning animal (Fig. 3 *C*, *Bottom*) in STILLNESS (blue). Two examples of the complexity of the pattern are as follows: 1) In T4, the second period of PUSH by the imaged animal is accompanied by a stable low value of ΔF/F until the competitor starts to RESIST, whereupon the mean activity rises dramatically; 2) in T5, the winning animal is in STILLNESS, whereupon, at 320 s, it shows a dramatic rise in mean activity which coincides with RESIST by the other animal and then subsides, with the trial ending through WITHDRAWAL of the loser. Thus, careful inspection reveals that perception of the social dimension was apparent even in this normalized multicell activity.

Fig. 3*D* shows the normalized mean overall neural activity (ΔF/F) for all successfully recorded animals. As in Fig. 2, the strong winners/losers (green) are considered separately from the moderate winners/losers. Mean activity is high in strong winners (a stable normalized score of circa 0.4 through the session) but much lower in the clear losers. Conversely, the mean activity patterns of the moderate subgroup (*n* = 6) are more equivalent with, if anything, the trend paradoxically showing losing animals to have slightly higher mean activity. Inhomogeneity of variance precluded the use of an ANOVAR, so we used a nonparametric test with the strong subgroup. These revealed that the difference in mean activity between winners and losers was highly significant in the case of strong dominance pairs (W = 0.39; L = 0.20; Mann–Whitney *U* = 1, df 23, *P* < 0.0001). The comparison of winners and losers in the moderate subgroup was not significant (W = 0.29; L = 0.33; Mann–Whitney *U* = 85, df 28, *P* > 0.3). Fig. 3*E* plots the normalized mean ΔF/F of individual animals considering winning trials and losing trials separately. The mean ΔF/F on winning trials was higher than on losing trials (paired samples *t* test *t* = 6.69, df = 130, *P* < 0.0001). The difference appears strongest in the contrast between strong winners and losers.

### Relationship of Cell Firing to Behavior.

**Fig. 4.**
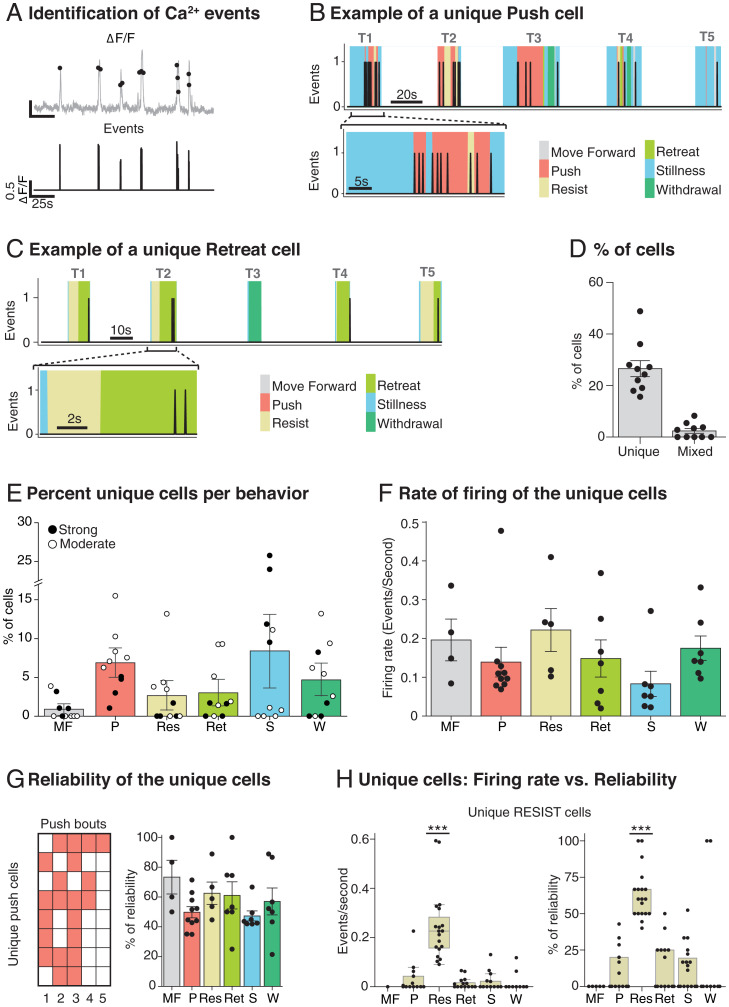
Identification of Ca^2+^ events and their correlation with behavior. (*A*) Event identification software takes the smoothed ΔF/F time signal (*Top*, gray); it identifies discrete events that cross a predefined threshold (*Top*, black dots) and time stamps these events (*Bottom*). (*B*) Exemplar of a unique PUSH cell activity during one session (*Top*) and in one expanded trial (*Bottom*). All the behaviors are represented with different colors. Note that the activity of the cell clearly overlaps with the PUSH behavior. (*C*) Exemplar of the distinct category of a unique RETREAT cell activity during one session (*Top*) and in one expanded trial (*Bottom*). (*D*) Percentage of recorded cells classified as unique (26.6% ± 3.1, *n* = 10) and mixed (2.4 % ±0.9, *n* = 10). (*E*) Percentages of cells classified as unique for one behavior. Note that the moderate animals (white dots) have more cells per behavior, except for STILLNESS (*n* = 10). (*F*) Rate of firing of the unique cells. Note that, despite STILLNESS being the most frequent behavior and having the highest number of specific cells, its mean firing rate is low. (*G*) Reliability of the unique cells inside a session. (*H*) Firing rate vs. reliability plots of the unique-RESIST cells across all the behaviors. Each session was treated independently with cells pooled across sessions. Note that the values are clearly higher for the behavior that they are encoding, which illustrates a complementary way of establishing the correct behavior classification of these cells (***P < 0.0001, *n* = 19). Means ±1 SEM.

**Fig. 5.**
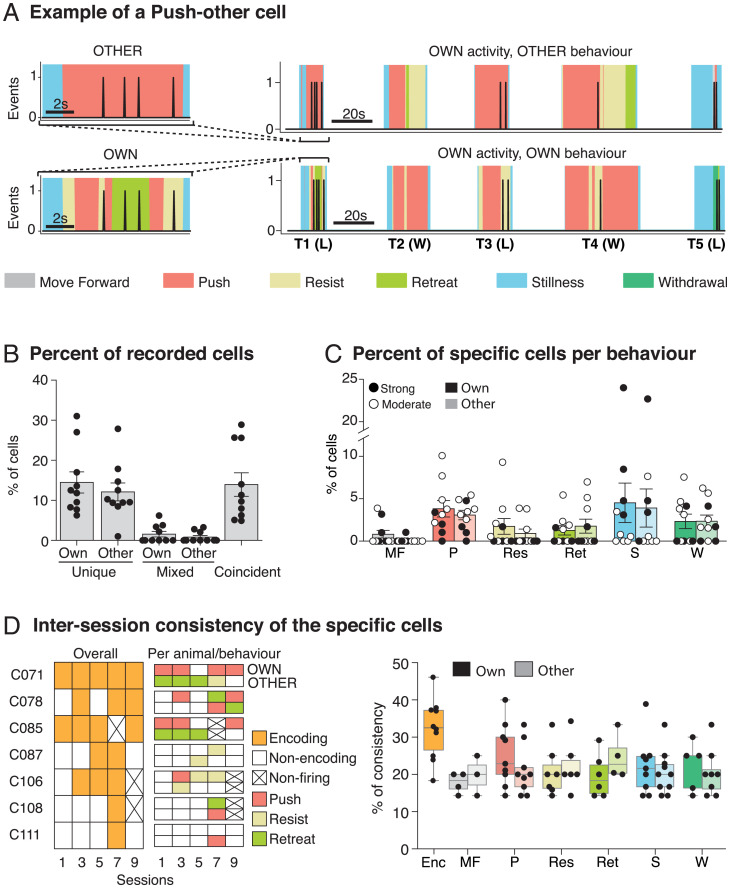
Identification of Ca^2+^ events and their correlation with the behavior of the other animal. (*A*) Exemplar of a PUSH-other cell activity across one session compared to the other animal behavior (*Top Right*) and the own animal behavior (*Bottom Right*) and expanded trials (*Left*) that show that the cell is active only when the other animal pushes (*Top*). Note that the imaged animal (H4089) is the winner (W) in T2 and T4 and is the loser (L) in T1, T3, and T5, since this rat is a moderate loser. (*B*) Percentages of recorded cells classified as specific for own, other, and coincident behaviors (*n* = 10). (*C*) Percentages of cells per behavior distinguishing between own and other behavior. Note that the percentages are quite similar between own and other and that the moderate dominance animals have more specific cells per behavior, except for STILLNESS (*n* = 10). (*D*) Consistency of the specific cells across sessions. (*Left, ’Per session. Example of a single rat’*) The overall consistency of seven exemplar cells from a single animal across five sessions (S1, S3, S5, S7 and S9) and the specific encoding per animal (OWN and OTHER) and behavior. (*Right, ’Per behavior’*) The overall consistency and the consistency per behavior. Note that the consistency of the encoding cells (30%, orange) and the consistency per behavior (20%, various colors) are much lower than the reliability values in Fig. 4 (*n* = 10). Means ±1 SEM.

Using Online Active Set method to Infer Spikes (OASIS) of ΔF/F signals, we inferred time-stamped activity corresponding to the sharp rise in amplitude that we hereafter consider as cell firing “events” (Fig. 4*A*; see *Materials and Methods*). Sessions were characterized by multiple time-stamped events across trials from which we computed a mutual information score (MIS) to identify individual cell activity that was uniquely associated with each of the six observed behaviors. A key finding was that unique cells were identified. Fig. 4*B* shows the event train for an exemplar unique PUSH cell (overlaid with corresponding behaviors during their time periods in distinct colors), with T1 time-expanded (below) to illustrate the close correlation between the activity of this cell and the act of pushing. Similarly, Fig. 4*C* shows the event train for an exemplar unique RETREAT cell throughout an exemplar five-trial session with, again, a clear correlation between the cell activity and the retreating behavior. Unique cells were observed in all six behavior categories, although those for STILLNESS cannot be interpreted. For each session, the MIS (bits) was computed between each cell event train and the six behaviors—a cell was classified as a unique behavior-specific cell if it superseded the 95th percentile of a randomly generated MIS distribution of shuffled activity with original behavior (*SI Appendix*, Fig. S1). This was computed independently for all six behaviors, with cells classified as unique for two behaviors in a single animal and categorized as “mixed cells” (Fig. 4*D*). The total number of unique behavior-specific cells was 262 at the 95% MIS criterion (26.6%) with 26 mixed cells (2.4%). There were a further 571 nonspecific cells (57.2%), the remainder being 13.9% coincident activity cells (Fig. 5; see *Materials and Methods*). There was no relationship to the dominance status of the animals (ANOVA F = 1.13, df 2/4, *P* = 0.41), or to the strength of this status (ANOVA F < 1). Fig. 4*E* reveals the proportion of unique cells across the six behavior categories with a relationship to the strength of the dominant status (ANOVA F = 9.27, df 5/10, *P* = 0.002). Fig. 4*F* shows the mean rate of firing (circa 0.15 events per second; computed as the number of events divided by the total time that unique behaviors were occurring; not significantly different across behaviors, F < 1). Reliability was measured by examining whether the activity of a unique cell occurred on all or subset of the behavioral bouts of a single session (Fig. 4*F*). Across all unique cells, reliability was relatively high (mean = 56.5%). With the identification of unique cells being a key finding, we show the example of unique RESIST cells with respect to both rate of firing and reliability. Note that, even with an MIS score of >95%, there were occasional events in those same cells during other behaviors, but in all cases these were rare; the firing rate was significantly higher for RESIST cells during resistance bouts compared to during other behaviors (Kruskal–Wallis *P* < 0.0001). Moreover, the reliability of the unique RESIST cells was circa 60%, whereas the reliability of these same cells during other behaviors was low (ANOVA F = 18.60, df = 5, *P* < 0.0001). Both results together illustrate the accuracy of the cell classification based on MIS.

Up until now, we have referred to unique cells as being those whose activity has an MIS score above the 95th percentile for a specific behavior. Fig. 5 introduces a new distinction concerning whether cells show a unique relationship to the animal’s own behavior or to that of the other animal. The tube test is, of course, a social situation; cell firing may be unique-own or unique-other. An exemplar of a unique-other cell is shown in Fig. 5*A*. This cell was recorded in one of the animals and fired only when the other animal pushed and went on to win in T1. A time expansion of T1 (Fig. 5 *A*, *Left*) shows this cell firing when the other animal pushed with the recorded loser sometimes resisting or retreating. This pair of animals was classified as showing only moderate dominance, with the recorded animal losing on T1, T3, and T5 but winning on T2 and T4 (Fig. 5*A*). We interpret the event train for this cell as one that uniquely encodes the other animal pushing. Fig. 5*B* shows the proportion of the different subsets of unique and mixed cells, but now including a further category called coincident cells, excluded from Fig. 4*D* for clarity. These were a further 124 cells (13.9%) that fired only when the behavior of animal A occurred simultaneously with a behavior of animal B. As shown in Fig. 5*C*, we did not observe any significant difference in the percentage of cells in each category related to the strength of the dominance status for either unique-own cells nor for unique-other cells (strength of status x category x behavior: ANOVA F < 1). However, dominance status did interact significantly (status*category*behavior: ANOVA F = 8.35, *P* = 0.002, df 5/10). The final measure calculated was intersession consistency of the two types of unique cells. Whereas reliability refers to cell/behavior correlations within a session, consistency is a measure of engagement across sessions with respect to encoding of any behavior (orange) or of a specific behavior such as PUSH, RESIST, or RETREAT. Fig. 5 *D*, *Left* gives examples for seven cells from one animal (H4011). Cell 71 is an example of high consistency across sessions (5/5) but in which the specific behavior encoded changes from session to session. In contrast, C87 shows lower consistency with respect to encoding any behavior (2/5 sessions). Fig. 5 *D*, *Right* shows the intersession consistency which was circa 30% for the overall encoding (Enc, orange) and 20% for the specific cells per behavior, both measures being much lower than for reliability.

## Discussion

There were two main findings. First, the mean cell activity (ΔF/F) across all cells in PrL of the mPFC of individual animals reflected relative dominance, being higher in strongly dominant and lower in highly submissive animals than in pairs of animals displaying more-moderate and interchanging patterns of dominance. Second, interanimal analyses revealed exacting MIS-computed correlations between Ca^2+^ activity in specific longitudinally registered regions of activity and specific behaviors. Unique cells were identified that correlated with an animal’s own behavior (unique-own) or the other animal’s behavior (unique-other). Coincident cells were also observed reflecting the joint behavior of the two animals competing in a tube test contest. We also made observations of within-session reliability and intersession consistency of the cell behavior correlations.

Detailed inspection of the data revealed that specific behaviors, such as pushing, resisting, and stillness, were frequent (Fig. 2). Retreat and withdrawal were more frequent in losing animals, but pushing was of a longer duration in very dominant winners. Stillness was also very frequent, but it is unclear what social engagement is happening during such periods. The focus of our analysis was on identifying whether there was cell activity in PrL that was uniquely correlated with these or other behavior categories, and the significance of the correlation assessed using a rigorous MIS (Figs. 4 and 5). Our findings of unique-own, unique-other, and coincident categories build on this concept, including that their relative frequency is reported and is surprisingly similar (Fig. 5*B*). That there are cells in PrL that are uniquely correlated to a dominant animal’s own pushing behavior is unsurprising given earlier observations using tetrode recording (10). However, we also observed 3% push-other cells that fired when the other animal was pushing (exemplar cell in Fig. 5*A*), and a smaller but significant number of RETREAT-other and withdraw-other cells (Fig. 5*C*). WITHDRAW-other cells are a particularly interesting category, as they are seen in winning animals when they were not engaged in any other behavior than stillness. The stationary winning animal could have only observed the losing animal withdraw. Our use of MIS scores above the 95th percentile criterion for significance also enabled us to separately identify coincident cells, namely, cells that fired only when one animal executed behavior A while the other animal was doing behavior B. The existence of these distinct categories supports our perspective that the activity of cells in PrL during social dominance encounters reflects a cognitive rather than a purely behavioral dimension of the situation.

Whereas single-cell recording has classically been examined in relation to the perception, location, actions, or decisions of an animal from which recordings are being taken (14–17), a social situation introduces the intriguing possibility that neural activity in animal A is responding to some facet of the behavior of animal B. With growing interest in social neuroscience, a wider facet of our results is that they constitute data having theoretical and methodological implications. In studies in nonhuman primates, the discovery of “mirror neurons” introduced the concept that activity in a motor area of the brain may be responsive to a specific action of the agent from which the recordings are being taken and to the same action undertaken by a different agent (19). The representation of the actions by others has also been observed in a study of social agency between two monkeys (20). In certain game-playing situations in which two monkeys cooperate in playing a game, patterns of single-cell firing have been observed in STS that correlate with acts of cooperation (21). Related to this, the concept of “theory of mind,” having to do with interpreting the intentions and actions of others, has also been intensively investigated in human neuropsychology and cognitive science, and in relation to ASDs (22).

Kingsbury et al. (11) were the first to report striking correlations of activity between the two animals in a social dominance situation, using the mean ΔF/F measure, leading to their statistical concept of interanimal “neural dynamics.” Such correlations might arise, as Kingsbury et al. themselves point out, because both animals receive a common exogenous stimulus at the same time (e.g., an auditory stimulus), but they conducted relevant controls to rule out such a “common-cause” explanation. They might also arise because the two animals are effortfully pushing against each other. We recognize and accept the concept of “interbrain dynamics” (12), but their behavior analyses did not appear to distinguish between pushing by one animal during resist by the other, because they used a common behavioral category of push for both. As we did not have two endoscopic cameras, we were unable to explore or extend their central finding, but we did manage to disentangle some correlations. For example, unique-own cells for pushing in dominant animals from which imaging was being done could be distinguished from different cells that were active in one animal that were also active during, for example, resist by the other animal (our coincident cells). The mean ΔF/F activity of the two brains would likely correlate very highly during this latter condition. Kingsbury et al. did take the precaution (notably, in their figure 7) of restricting their consideration to cases in which one animal was motionless and the other animal did something. This is suitably cautious but may not be the only approach to disentangling cell firing related to self vs. other with, for example, our distinction between unique-other and coincident cells. An additional value of the behavior categorization of individual cells is that it enabled us to ask about the reliability of cell firing during an individual session, and the consistency from session to session. Our data suggest that PFC encoding is dynamic, with consistency low (circa 33% across multiple sessions) and lower for specific behaviors.

We began this program of research to investigate possible disorders in social dominance in rat models of ASDs, finding, as expected, that both Fragile-X and SynGap mutant rats are socially submissive but with dominance patterns influenced by experience (6, 23). In seeking to take this project to a neurophysiological level, we are now seeking to enlarge the cohort of animals to be able to compare Ca^2+^ signals in these different lines with those of WT normal rats. This analysis will be complemented by studies in other social protocols such as sociability and social memory, both of which are relevant to a better understanding of the neurobiology of ASDs.

## Materials and Methods

Contact for Reagents and Resources Sharing.

Further information and requests for resources and reagents should be directed to and will be fulfilled by the lead contact, R.G.M.M. (r.g.m.morris@ed.ac.uk).

### Materials Availability.

This study did not generate new unique reagents.

### Data and Code Availability.

The data are available at https://datashare.ed.ac.uk/handle/10283/4373, and consists of Excel files corresponding to each panel of each figure. The code to classify behaviorally tuned neurons is available at https://github.com/rufusmitchellheggs/Behavioral-Tuned-Cells.

### Experimental Model and Subject Details.

#### Rats.

Adult (*n* = 12) male Long-Evans Fmr1^em1/PWC^ hooded rats, hereafter referred to as WT or Fmr1 KO, were used, aged 3 mo to 4 mo at the start of surgery, weighing circa 350 g. The rats were housed in groups of two per cage, with ad libitum food/water and a 12-h light/dark cycle. The Fmr1 KO rats were generated by mating female heterozygous rats with male WT Long-Evans hooded rats obtained from Sigma Advanced Genetic Engineering (SAGE) laboratories, now part of Horizon Discovery, and the offspring of the female Fmr1 heterozygotes crossed to WT Long-Evans hooded rats (Charles River Labs), thus FMRP^-/Y^. The WT rats were littermate matched. There were three WT cages with *n* = 2 per cage, and three Fmr1 KO cages (ns = 2). All experiments were done blind to genotype, with animals being given a colored spot on their fur (using animal paint) and their cage number on their tail to identify them. The code was retained by someone independent of the study.

#### Ethical and legal issues.

All procedures related to animals, including surgical procedures, involved a series of stages, each conducted under recovery anesthetic (isoflurane), according to the regulations of the Animals (Scientific Procedures) Act 1986 and under the supervision of the named veterinary surgeons of the University of Edinburgh.

### Surgical Methods.

#### Virus injection.

The animals were anesthetized using isoflurane (1 to 3%) and mounted in a stereotaxic apparatus (Kopf). A hole was drilled in the skull, targeting the prelimbic (PrL) region of the mPFC at stereotaxic coordinates AP −3.2 mm, ML 0.8 mm (unilateral infusion), and DV at both 3.7 and 4.2 mm. The virus, AAV1.CamKII.GCaMP6f.WPRE.SV40 (source: https://www.addgene.org) diluted with saline to a final dilution of 1:5 (titer: 5.77 × 10^12^ GC/mL), was infused using the 30G needle and a nanoinjector pump (previous virus dilution studies were conducted to establish an optimal dilution for this brain region). A volume of 250 nL per site was infused at the rate of 50 nL/min (5 mins). A further delay of 5 min to allow the virus to diffuse was allowed before the needle was retracted slowly. Beginning with an immediate postoperative injection of rimadyl, the animals were allowed to recover for 2 wk, with postoperative care undertaken to minimize the discomfort to the animal as per institutional guidelines.

#### GRIN lens implantation.

Three to four weeks after virus injection, the GRIN lens was implanted at the same stereotaxic location. The animals were again anesthetized and fixed in the stereotaxic apparatus. Using a trephine bur to do a very small craniotomy above the virus injection site, followed by the creation of a small track cut into the brain tissue overlying PrL by lowering a sterile blunt needle (18G) through the hole in the brain at the target site (DV 3.25 mm from skull surface), we prepared for lowering the GRIN lens. When the needle reached its target site, we paused for 5 min, and then the needle was retracted slowly; this created a cavity which was almost the size of the lens (1-mm diameter). Using a holder assembly, the GRIN lens was lowered into the brain slowly until it reached a site 3.25 mm below the skull surface. Dental cement (Super Bond) was used to secure the lens to the skull and four skull screws that had previously been drilled into location as solid anchor points. The lens was then covered with silicon to protect it until the final step of baseplate implantation.

#### Baseplate implantation.

A period of ∼3 wk after lens implantation is required for the lens to become clear (clearance of implantation-associated gliosis). Using anesthesia again, the baseplate containing a working endoscope was lowered so as to target the center of the GRIN lens. We followed Inscopix guidelines to lower the baseplate until the circular border of the lens is seen, and then moved up slightly until we saw cells and/or blood flow, whereupon, at this point, we used dental cement to fix the baseplate. When the cement had dried and hardened, the scope was removed gently by undoing the baseplate screw, leaving the baseplate with the GRIN lens. The baseplate was then covered with the baseplate cover.

#### Protecting the endoscope.

Due to the social nature of our behavioral task, we find that the endoscope needs protection from the other animal to secure stable Ca^2+^ recordings. We used our three-dimensional (3D)-printed protector [called iHELMET; see Saxena et al. (18)]. Briefly, an additional plastic baseplate surround is secured permanently on the animal 1 d after the baseplate installation, care being taken to align it with the baseplate in such a way that, when the endoscope is mounted and the iHELMET is placed, it protects the endoscope without any direct contact with the endoscope. A cable clip on the helmet also prevents any sharp movements of the cable from causing force on the camera to which it is electrically connected.

### Apparatus.

#### Behavior.

The tube test assay was procedurally as in Saxena et al. (6). A 1-m transparent Plexiglas tube, 7-cm internal diameter, served to connect two holding boxes (42 × 26 × 18 cm; Fig. 1*D*). In each box, bedding was placed from the home cage of the animals to help reduce anxiety. The tube was large enough for the rats to move freely, but to neither cross each other nor turn around. The apparatus was modified from that of Saxena et al. (6) to include 1) a long slit cut into the upper central part of the Plexiglas to permit lateral movement of the head-mounted endoscope and attached cable along the axis of the tube; and 2) a moveable wire-grill separator that permitted visual, auditory, and olfactory interaction of the animals in the tube but limited somatosensory contact, running on a smoothly running trolley such that the slightest movement onto it from either animal would result in it moving effortlessly. A camera provided a direct view of the tube to record the trials using OBS recording software. The entire apparatus was connected to custom-made Arduino-based hardware, and we used its serial reader functionality for reading the button press (start/stop times of the trials) into the computer.

#### Calcium imaging.

The Inscopix data acquisition software and Inscopix nVista imaging system (v3.0) associated with a GRIN lens are used to acquire calcium imaging videos at 20 Hz (24, 25). The procedure for implanting the virus and GRIN lens is described above. The endoscopic camera was mounted daily in exactly the same place on a head-fixed baseplate and connected, via a power/data cable, to a data receiver and then to relevant computers. Recordings were stabilized and the camera was protected using the “iHELMET” (18). During each trial, the experimenter was located in an antechamber separate from the animals to limit human–animal interference.

### Procedure.

The training protocol consisted of habituation followed by tube test contests between animals within each cage coupled to Ca^2+^ imaging (Figs. 1*E* and 2*A*).

#### Habituation.

One week prior to the start of the contests, small sections of the Plexiglas tubing were placed inside each home cage, and the animals were handled daily. The two animals per cage then received their color marking, and were placed into the apparatus for 20 min. Both cage occupants were allowed to run freely through the tubes for 5 min before being returned to the home cage.

#### Tube test contests.

A tube test contest consisted of two rats being placed in the holding boxes, one on each side. Both rats had an iHELMET (red or green) of which one contained a working camera and the other a dummy camera. Recordings were taken from both rats on alternate sessions (1, 3, 5, etc. and 2, 4, 6, etc.; we had only one endoscope). The rats then entered the tube and met in the middle at the metal grid which acted as a barrier. The trial started when the moveable barrier was unlocked. During the trial, the rats competed for dominance, during which a variety of behaviors were observed (identified blind with respect to genotype). Typically, the animals were together and relatively still for a few seconds. Thereafter, either one rat pushed the subordinate out of the tube (dominant) or the other rat withdrew of its own accord (subordinate), but various other behaviors occurred during such encounters. MOVE FORWARD occurred when either or both animals moved toward each other; PUSH occurred when animal A pushed against the metal grid and caused it to move, often stopped by animal B showing RESIST behavior. The roles of animals A and B in executing PUSH and RESIST generally alternated, but not exactly in time with each other. If animal A was showing PUSH behavior and animal B being pushed backward, this was called RETREAT (i.e., an enforced action); however, if animal B withdrew of its own accord, this was classified as WITHDRAWAL (i.e., an unenforced action). There were sometimes periods when neither animal moved or did much else; these were classified as STILLNESS, but this could include sniffing, grooming, or other activities that do not involve forward or backward motion. A trial was defined as ending when the first rat retreated or withdrew into the holding box from which it started. This rat was recorded as the “loser,” and the other was recorded as the “winner.” Each pair of rats underwent five trials each session, alternating their starting positions from left or right, to obtain a secure measure of dominance (5:0 wins/losses, 4:1 or 3:2). One session of each of 10 sessions was completed each day. Randomization of the pair sequence and cage sequence was applied so that both changed every session. Trial latency was taken as the time (seconds) to complete one trial. All trials included both behavioral observation and Ca^2+^ imaging. Each animal had an iHELMET, but only one animal had a real nVista, the other wore a “dummy” camera (the camera is expensive). The animal from which recordings were being taken alternated across days. Video recordings were taken at the same time.

### Data Analysis.

#### Behavioral labeling.

The data analysis proceeded in stages. With respect to behavior, the videos of all four animals were analyzed using BORIS software (26), with the occurrence of six different categories of behavior examined on a frame-by-frame basis and time stamped to 1/20-s resolution (the same as the frame rate of the Inscopix camera). Thus, we knew when each behavior began and ended to that temporal resolution.

#### Single-photon calcium imaging.

For Ca^2+^ recordings, the field of view was preprocessed by first applying a low and high spatial band-pass filter (σ_low_ = 0.0005 and σ_high_ = 0.5), then motion correcting by aligning each frame to a manually selected high-activity reference frame (both algorithms were used within the Inscopix data processing software [IDPS, Inscopix (27)]. The subsequent preprocessed recordings were then exported as a .tiff file, cell ROIs were extracted, and their respective ΔF/F were computed using the CNMF-e python API (28). Cells were longitudinally registered across multiple sessions by reading the CNMF-e identified ROIs into the Inscopix IDPS environment and using their internal registration algorithm to align ROIs across days. Finally, event detection was computed using the OASIS package (29) (as found in CaImAn). Care was taken to use individual animal noise thresholds (stable across sessions) to identify events. A low threshold risks assigning noise to “events,” whereas a high threshold misses “events” (typically, we adapted the “s_min” parameter to be between 0.2 and 0.3). We identified an appropriate threshold to optimize the signal-to-noise ratio for each cell in each animal and maintained that threshold across all longitudinally registered sessions (the threshold changing to 0.1 and others at 0.4 in rare cases). Having identified Ca^2+^ events, and time stamped them, it was then possible to align the behavior and Ca^2+^ time series exactly. Visual inspection of output data was used to verify correct performance of the cell identification and event detection algorithms.

#### Behaviorally tuned neurons.

Each neural event train was convolved with a Gaussian function (σ = 12.5 ms, window width of 2 σ) to obtain a time series of instantaneous firing rates. The Kraskov MIS was then calculated independently between each neuron and each behavior (30). Neurons were classified as encoding a specific behavior during a session (i.e., five trials) if they met the following criteria: 1) Calcium transient events were present in over 34% of the behavioral occurrences, and 2) the cell achieved an MIS greater than chance. Chance-level mutual information for a cell was determined by performing 2,000 shuffles of a cell event train and calculating the mutual information between each shuffled event train. The cell was considered as encoding a specific behavior cell if its MIS exceeded the 95th percentile of the values for the shuffled data. All the cells classified as encoding a specific behavior were verified with a visual inspection.

#### Behaviorally tuned neuron characteristics.

For all behavioral encoding neurons, the “reliability” was calculated as the percentage of within-session behavioral occurrences that a neuron fired, and their “consistency” was calculated as the percentage of sessions in which the neuron was classified as encoding a specific behavior. The event rate was calculated as the number of events divided by the duration of behavior (seconds) to yield events per second—these were then averaged across behavioral occurrences and across all sessions.

### Terminology.

We followed standard usage in Ca^2+^ imaging studies for defining specific terms. When the camera displays a Ca^2+^ transient, we refer to this as an event, and events occur at a certain rate. Such transients happen in an ROI on the screen image of the endoscope, and these ROIs are considered as cells. Thus, events are cell firing. Longitudinal registration with respect to the *x*,*y* location of the ROI, identified via the stable physical location of ROIs in the camera image (31), enables a single cell to be followed across days (Fig. 3*A*). Events may occur at a single cell only during specific behaviors in the tube test (self or other or both), or during multiple behaviors, and thus their categorization was into unique cells which show specific events only during one behavior, coincident cells (unique behaviors in one animal of a pair simultaneously with a unique behavior in the other animal), mixed cells (any two behaviors in a single animal), or nonspecific cells (three or more behaviors). We distinguished unique-own, unique-other, and coincident cells. We also measured rate of firing (events per unit of time that a specific behavior was happening), and so could measure separately the number of cells by behavior category and the rate of events per category.

The critical feature of our correlational methodology was the principle of “mutual information.”

### Histology and Histochemistry.

At the end of all recording, the animals were perfused transcardially with cold PBS (phosphate buffer saline, P4417 Sigma Aldrich) and fixed using 4% formaldehyde in PBS (prepared from Paraformadehyde, Sigma Aldrich 441244). After cryopreservation in 20% sucrose, coronal sections (60 µm thick) were cut on a cryostat and then visualized (endogenous GCaMP and staining signal) using a Nikon A1R confocal microscope. In all animals, the lens was targeted correctly to PrL as identified from the Rat Brain Atlas. For immunofluorescence staining, slices were permeabilized in PBS 10% NDS (normal donkey serum, Sigma Aldrich D9663) 0.3% Triton X-100 (Sigma Aldrich T8787) for 30 min, then incubated in PBS 10% NDS 0.1% Triton X-100 with 1:1,000 mouse Anti-GAD67 Antibody (Sigma Aldrich MAB5406 clone 1G10.2) or 1:500 guinea pig Anti-NeuN (SYSY 266 004) overnight at room temperature. The next day, three 10-min washes with PBS 0.1% Triton X-100 were performed, then slices were incubated in PBS 0.1% Triton with 1:200 donkey anti-mouse Alexa 647 (Thermo Fisher A-31571) or 1:200 goat anti-guinea pig Cy3 (Abcam ab102370), After three washes in PBS, slices are mounted in Fluoroshield with DAPI (Sigma Aldrich F6057). Confocal images were acquired with air objective 20× Plan Apo VC/NA 0.8, and the pinhole was set to 1.5 AU; 405-, 488-, 561-, and 633-nm laser lines were used to acquire the DAPI, GCaMP, Cy3, and Alexa 647 channels, respectively. Image processing was done with ImageJ (NIH).

### Quantification and Statistical Analysis.

Standard statistical tests were used, as data demanded, with the SPSS statistical package v25 used for these tests. Statistical significance was defined with α < 0.05, with all F values and degrees of freedom stated. Specifications were described in the respective results and figure legends. All of the bar graphs with error bar represent mean ± SEM. Line graphs with error bars represents mean ± SEM.

## Supplementary Material

Supplementary File

## Data Availability

Excel files and Python code have been deposited at https://datashare.ed.ac.uk/handle/10283/3888. The code to classify behaviorally tuned neurons is available at https://github.com/rufusmitchellheggs/Behavioral-Tuned-Cells.

## Change History

August 1, 2022: The author byline has been updated to correct an author name misspelling.

## References

[1] L. Ricceri, A. Moles, J. Crawley, Behavioral phenotyping of mouse models of neurodevelopmental disorders: Relevant social behavior patterns across the life span. Behav. Brain Res. 176, 40–52 (2007). 16996147 10.1016/j.bbr.2006.08.024

[2] J. N. Crawley, What’s Wrong with My Mouse? Behavioral Phenotyping of Transgenic and Knockout Mice (Wiley, Hoboken, NJ, ed. 2, 2006).

[3] G. Lindzey, H. Winston, M. Manosevitz, Social dominance in inbred mouse strains. Nature 191, 474–476 (1961). 13762409 10.1038/191474a0

[4] Z. Fan , Using the tube test to measure social hierarchy in mice. Nat. Protoc. 14, 819–831 (2019). 30770887 10.1038/s41596-018-0116-4

[5] F. Wang , Bidirectional control of social hierarchy by synaptic efficacy in medial prefrontal cortex. Science 334, 693–697 (2011). 21960531 10.1126/science.1209951

[6] K. Saxena , Experiential contributions to social dominance in a rat model of fragile-X syndrome. Proc. Biol. Sci. 285 (2018). 10.1098/rspb.2018.0294 PMC6015851 29899064

[7] A. Asiminas , Sustained correction of associative learning deficits after brief, early treatment in a rat model of Fragile X Syndrome. Sci. Transl. Med. 11 (2019). 10.1126/scitranslmed.aao0498 PMC8162683 31142675

[8] J. Guy, J. Gan, J. Selfridge, S. Cobb, A. Bird, Reversal of neurological defects in a mouse model of Rett syndrome. Science 315, 1143–1147 (2007). 17289941 10.1126/science.1138389 PMC7610836

[9] A. J. Sandweiss, V. L. Brandt, H. Y. Zoghbi, Advances in understanding of Rett syndrome and MECP2 duplication syndrome: Prospects for future therapies. Lancet Neurol. 19, 689–698 (2020). 32702338 10.1016/S1474-4422(20)30217-9

[10] T. Zhou , History of winning remodels thalamo-PFC circuit to reinforce social dominance. Science 357, 162–168 (2017). 28706064 10.1126/science.aak9726

[11] L. Kingsbury , Correlated neural activity and encoding of behavior across brains of socially interacting animals. Cell 178, 429–446.e16 (2019). 31230711 10.1016/j.cell.2019.05.022 PMC6625832

[12] L. Kingsbury, W. Hong, A multi-brain framework for social interaction. Trends Neurosci. 43, 651–666 (2020). 32709376 10.1016/j.tins.2020.06.008 PMC7484406

[13] F. L. Hitti, S. A. Siegelbaum, The hippocampal CA2 region is essential for social memory. Nature 508, 88–92 (2014). 24572357 10.1038/nature13028 PMC4000264

[14] D. H. Hubel, T. N. Wiesel, Receptive fields, binocular interaction and functional architecture in the cat’s visual cortex. J. Physiol. 160, 106–154 (1962). 14449617 10.1113/jphysiol.1962.sp006837 PMC1359523

[15] J. O’Keefe, J. Dostrovsky, The hippocampus as a spatial map. Preliminary evidence from unit activity in the freely-moving rat. Brain Res. 34, 171–175 (1971). 5124915 10.1016/0006-8993(71)90358-1

[16] E. V. Evarts, Brain mechanisms in movement. Sci. Am. 229, 96–103 (1973). 4198738 10.1038/scientificamerican0773-96

[17] M. N. Shadlen, W. T. Newsome, Neural basis of a perceptual decision in the parietal cortex (area LIP) of the rhesus monkey. J. Neurophysiol. 86, 1916–1936 (2001). 11600651 10.1152/jn.2001.86.4.1916

[18] K. Saxena, P. A. Spooner, R. Mitchell-Heggs, R. G. M. Morris, iHELMET: A 3D-printing solution for safe endoscopic Ca^2+^ recording in social neuroscience. J. Neurosci. Methods 355, 109109 (2021). 33705854 10.1016/j.jneumeth.2021.109109

[19] G. Rizzolatti, L. Fogassi, V. Gallese, Mirrors of the mind. Sci. Am. 295, 54–61 (2006). 17076084 10.1038/scientificamerican1106-54

[20] K. Yoshida, N. Saito, A. Iriki, M. Isoda, Representation of others’ action by neurons in monkey medial frontal cortex. Curr. Biol. 21, 249–253 (2011). 21256015 10.1016/j.cub.2011.01.004

[21] R. V. Bretas, M. Taoka, H. Suzuki, A. Iriki, Secondary somatosensory cortex of primates: Beyond body maps, toward conscious self-in-the-world maps. Exp. Brain Res. 238, 259–272 (2020). 31960104 10.1007/s00221-020-05727-9 PMC7007896

[22] F. G. Happé, Communicative competence and theory of mind in autism: A test of relevance theory. Cognition 48, 101–119 (1993). 8243028 10.1016/0010-0277(93)90026-r

[23] E. Harris , Experiential modulation of social dominance in a SYNGAP1 rat model of ASD. Eur. J. Neurosci. 54, 7733–7748 (2021). 34672048 10.1111/ejn.15500 PMC7614819

[24] B. A. Flusberg , High-speed, miniaturized fluorescence microscopy in freely moving mice. Nat. Methods 5, 935–938 (2008). 18836457 10.1038/nmeth.1256 PMC2828344

[25] E. J. Hamel, B. F. Grewe, J. G. Parker, M. J. Schnitzer, Cellular level brain imaging in behaving mammals: An engineering approach. Neuron 86, 140–159 (2015). 25856491 10.1016/j.neuron.2015.03.055 PMC5758309

[26] O. Friard, M. Gamba, BORIS: A free, verstaile open-source event-logging software for video/audio coding and live observations. Methods Ecol. Evol. 7, 1325–1330 (2016).

[27] E. A. Mukamel, A. Nimmerjahn, M. J. Schnitzer, Automated analysis of cellular signals from large-scale calcium imaging data. Neuron 63, 747–760 (2009). 19778505 10.1016/j.neuron.2009.08.009 PMC3282191

[28] P. Zhou , Efficient and accurate extraction of in vivo calcium signals from microendoscopic video data. eLife, 7 e28728 (2018). 29469809 10.7554/eLife.28728 PMC5871355

[29] J. Friedrich, P. Zhou, L. Paninski, Fast online deconvolution of calcium imaging data. PLOS Comput. Biol. 13, e1005423 (2017). 28291787 10.1371/journal.pcbi.1005423 PMC5370160

[30] A. Kraskov, H. Stögbauer, P. Grassberger, Estimating mutual information. Phys. Rev. E Stat. Nonlin. Soft Matter Phys. 69, 066138 (2004). 15244698 10.1103/PhysRevE.69.066138

[31] L. Sheintuch , Tracking the same neurons across multiple days in Ca^2+^ imaging data. Cell Rep. 21, 1102–1115 (2017). 29069591 10.1016/j.celrep.2017.10.013 PMC5670033

