# Supplementary material for: Ca2+ imaging of self and other in medial prefrontal cortex during social dominance interactions in a tube test
Source: Proc Natl Acad Sci U S A. 2022 Jul 26;119(31):e2107942119. doi: 10.1073/pnas.2107942119 (PMC9353509; doi:10.1073/pnas.2107942119)
Supplement: Supplementary File [file pnas.2107942119.sapp.pdf]

A. The identification of specific cells

Behaviour	Cell X
Push	0.663
Push	0
Push	0.471
Push	0.534
Resistance	0
Resistance	0
Retreat	0
Retreat	0
Push	0.778
Push	0.526
Push	0
Stillness	0
…	…

Behaviour	Cell X
Behaviour	Active
Behaviour	Not active
Behaviour	Active
Behaviour	Active
No behaviour	Not active
No behaviour	Not active
No behaviour	Not active
No behaviour	Not active
Behaviour	Active
Behaviour	Active
Behaviour	Not active
No behaviour	Not active
…	…

Behaviour	Cell X
1	1
1	0
1	1
1	1
0	0
0	0
0	0
0	0
1	1
1	1
1	0
0	0
…	…

Calculation of the **k** value as an average of 100 estimations

2000 shuffles

Behaviour	Cell X
1	0
1	0
1	0
1	0
0	1
0	1
0	1
0	0
1	0
1	1
1	0
0	1
…	…

MI_1

Behaviour	Cell X
1	0
1	1
1	0
1	0
0	0
0	1
0	0
0	1
1	1
1	1
1	0
0	0
…	…

MI_2

Behaviour	Cell X
1	1
1	1
1	1
1	1
0	1
0	0
0	0
0	0
1	0
1	0
1	0
0	0
…	…

MI_3

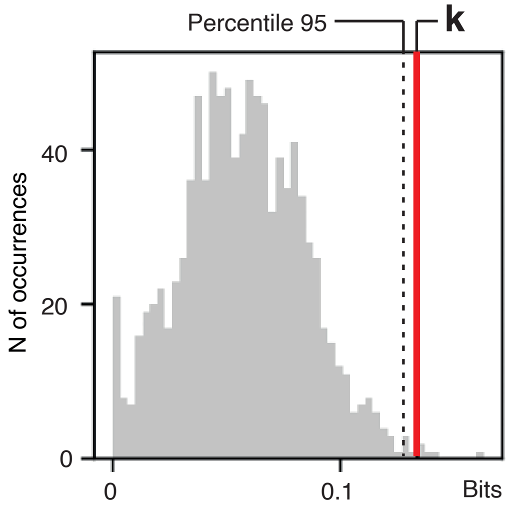

B. MI score and firing pattern

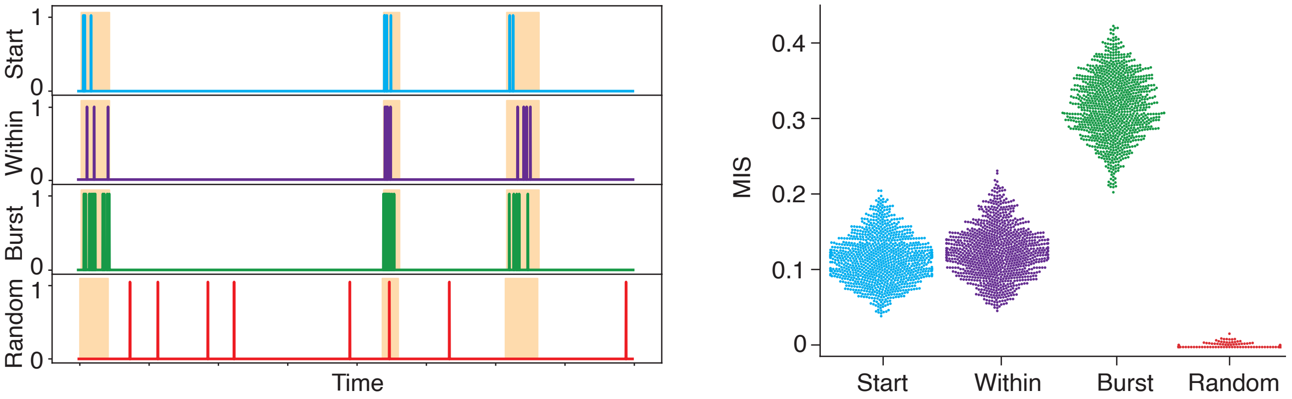

Supplementary Figure 1: *Identification of specific cells using the Mutual Information Score.* A) The algorithm transforms the lists of behaviors and the cell activity into binary and calculates the Mutual Information value (k) as an average of 100 estimations. After that, it randomizes the events of the cell 2000 times and calculates again the MI value. After doing the shuffles and obtaining the MI values, the algorithm creates a histogram with the MI values and their frequency. If the k value is higher than the 95th percentile, this cell can be considered as specific for this behavior. B) Simulation of cells with different firing pattern to see the variability of the MIS. Note that the cells that are more active during the beginning of the behavioral bout and the cells that are active randomly during the behavioral bout have similar MIS. However, the cells that have more events inside the behavioral bouts have higher MIS. Finally, the cells with a random pattern of activity have a MIS close to 0.
